# Characterization of two lytic bacteriophages, infecting *Streptococcus bovis*/*equinus* complex (SBSEC) from Korean ruminant

**DOI:** 10.1038/s41598-023-36306-x

**Published:** 2023-06-05

**Authors:** Seon Young Park, Hyemin Kwon, Sang Guen Kim, Se Chang Park, Ji Hyung Kim, Seongwon Seo

**Affiliations:** 1 Division of Animal and Dairy Sciences, College of Agriculture and Life Science, Chungnam National University, Daejeon, 34134 South Korea; 2 Department of Microbiology and Molecular Biology, College of Bioscience and Biotechnology, Chungnam National University, Daejeon, 34134 South Korea; 3 Laboratory of Aquatic Biomedicine, College of Veterinary Medicine and Research Institute for Veterinary Science, Seoul National University, Seoul, 08826 South Korea; 4 Department of Food Science and Biotechnology, College of Bionano Technology, Gachon University, Seongnam, 13120 South Korea

**Keywords:** Microbiology, Genomics

## Abstract

*Streptococcus bovis/equinus* complex (SBSEC) is one of the most important lactic acid-producing rumen bacteria causing subacute ruminal acidosis. Despite the significance of the ruminal bacteria, lytic bacteriophages (phages) capable of infecting SBSEC in the rumen have been rarely characterized. Hence, we describe the biological and genomic characteristics of two lytic phages (designated as vB_SbRt-pBovineB21 and vB_SbRt-pBovineS21) infecting various SBSEC species, including the newly reported *S*. *ruminicola*. The isolated SBSEC phages were morphologically similar to *Podoviridae* and could infect other genera of lactic acid-producing bacteria, including *Lactococcus* and *Lactobacillus*. Additionally, they showed high thermal- and pH-stability, and those characteristics induce strong adaptation to the ruminal environment, such as the low pH found in subacute ruminal acidosis. Genome-based phylogeny revealed that both phages were related to *Streptococcus* phage C1 in the *Fischettivirus*. However, they had a lower nucleotide similarity and distinct genomic arrangements than phage C1. The phage bacteriolytic activity was evaluated using *S*. *ruminicola*, and the phages efficiently inhibited planktonic bacterial growth. Moreover, both phages could prevent bacterial biofilms of various SBSEC strains and other lactic acid-producing bacteria in vitro. Thus, the newly isolated two SBSEC phages were classified as new *Fischettivirus* members and could be considered as potential biocontrol agents against ruminal SBSEC bacteria and their biofilms.

## Introduction

*Streptococcus bovis*/*Streptococcus equinus* complex (SBSEC) is a commensal bacterial group in the gastrointestinal tract of humans and domesticated animals, including cows, goats, and horses^1^. The SBSEC group has been classified based on phenotypic characteristics of the species, including *Streptococcus* (*S*.) *equinus* (synonymous with *S. bovis*), *S. gallolyticus* subsp. *galloyticus*, *S. gallolyticus* subsp. *pasteurianus*, *S. gallolyticus* subsp. *macedonicus*, *S*. *infantarius* subsp. *infantarius*, *S*. *lutetiensis*, and *S. alactolyticus*^2^. Recently, a new species, *S*. *ruminicola,* isolated from ruminants in South Korea, was proposed as a new member with distinct biological properties among strains of this group^3^. The specific (sub)species of the SBSEC have caused clinical infections, such as infective endocarditis and bacteremia with colorectal cancer. They are also involved in animal metabolic disorders, such as subacute ruminal acidosis in ruminants^4–6^, thereby indicating the significance of this group as potential pathogens.

Bacteriophages (phages) naturally infect bacteria through two replication lifecycles (lytic and lysogenic) and have high specificity host targeting, avoiding microbiome community disturbances^7^. The unique attributes of phages make them potential antimicrobials for selectively controlling several pathogenic bacteria. Bacterial biofilm consisting of extracellular polymeric substances is highly resistant to antibiotics, heat, and acidic conditions, resulting in increased antimicrobial-resistant bacteria in abiotic and biotic communities^8^. Phages have been reported to control biofilm formation and penetrate the existing biofilm on the surfaces of planktonic cells^9^. The phages have been found in the rumen at approximately 10^8^ particle populations per gram of rumen contents^10^, yet little is known in terms of the biological properties of most phages under strict cultivation environments. Until now, 14 phages infecting *Streptococcus* spp. were taxonomically approved by the International Committee on Taxonomy of Viruses (ICTV; https://ictv.global/taxonomy), and approximately 800 completely sequenced genomes, including *Streptococcus* phages, are available in the GenBank database. However, studies on phages infecting SBSEC are relatively scarcer than those of other *Streptococcus* sp. despite the importance of ruminal bacteria. Only one genome of the SBSEC phage isolate ϕSb01, with morphotypes of the *Siphoviridae* family, is currently available in the Joint Genome Institute Genome Portal^11^.

Nevertheless, SBSEC phages are one of the well-studied phages among the viruses infecting ruminal bacteria, such as *Ruminococcus albus* of the *Myoviridae* family^12^, and *Bacteroides* sp. of the *Podoviridae* family^13^. In addition, several lytic or lysogenic SBSEC phage isolates have been morphologically and genetically characterized^14–17^. Moreover, the possible application of the prophage-based endolysin from a sequenced SBSEC genome to control rumen microbiota has been reported^18^. However, to the best of our knowledge, SBSEC phages with morphotypes of the *Podoviridae* family have not yet been reported. Therefore, we report two lytic phages capable of infecting various ruminal SBSEC bacteria including the newly reported *S. ruminicola* and other genera of lactic acid-producing bacteria (e.g., *Lactococcus* and *Lactobacillus*) with strong potential for biotechnological applications. The biological and genomic characteristics of both SBSEC phages (designated as vB_SbRt-pBovineB21 and vB_SbRt-pBovineS21) and their ability to control bacterial biofilms were examined. This is, to the best of our knowledge, the first report of phages with *Podoviridae* morphotypes, which infect SBSEC species and other lactic acid-producing bacteria found in the rumen of ruminants.

## Results

### Isolation, morphology, and lytic spectrum of the isolated SBSEC phages

**Figure 1:**
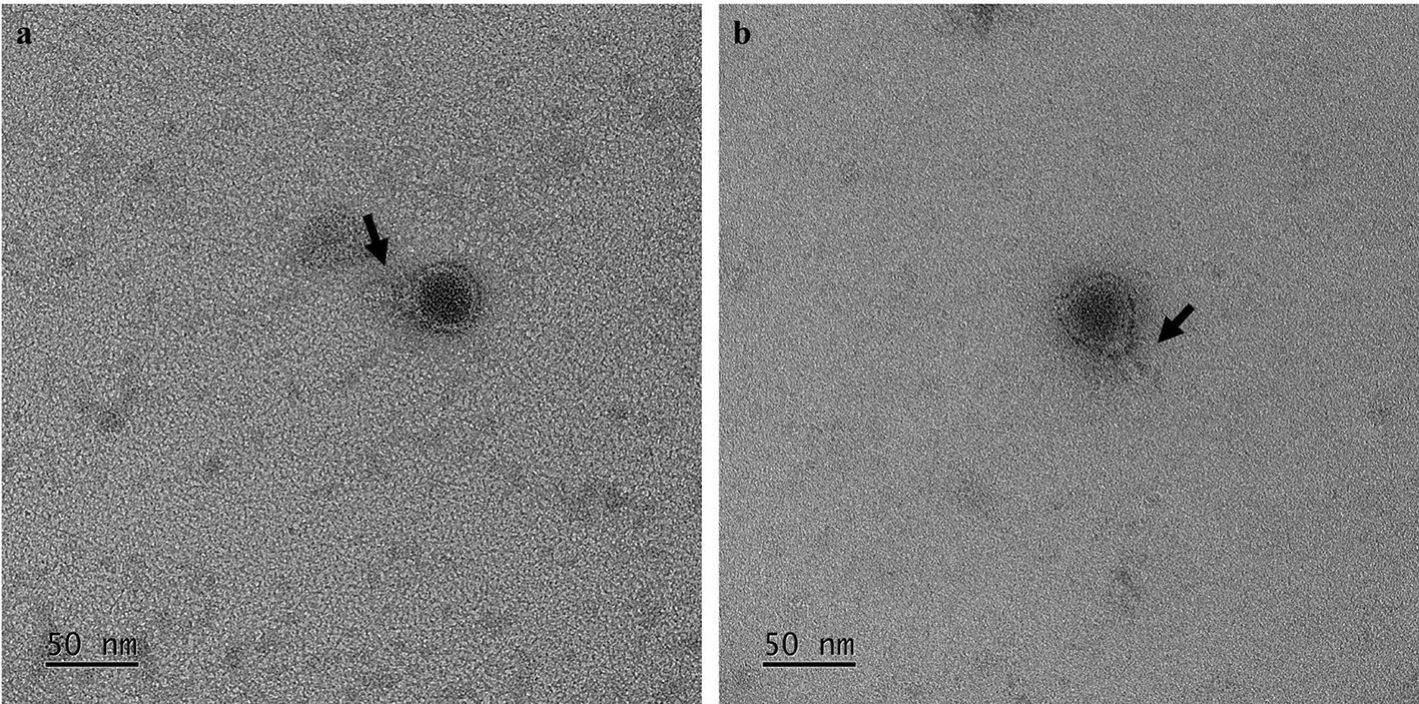
Transmission electron micrographs of the isolated SBSEC phages. (**a**) TEM image of vB_SbRt-pBovineB21phage showing an icosahedral head (49.2 nm) and a short non-contractile tail (13.3 nm) is indicated by a black arrow. (**b**) TEM image of vB_SbRt-pBovineS21 phage showing an icosahedral head (46.2 nm) and a short non-contractile tail (12.5 nm) is indicated by a black arrow. The scale bar is 50 nm.

A total of two SBSEC phages were isolated in this study, and these were designated as vB_SbRt-pBovineB21 and vB_SbRt-pBovineS21. SBSEC phage vB_SbRt-pBovineB21 was isolated from fecal samples collected from a Hanwoo farm (Chungcheongnam-do, Korea) and vB_SbRt-pBovineS21 was isolated from sewage samples obtained from a sewage treatment plant (Daejeon, Korea). Using the double-layer agar method, both isolated phages were able to produce clear plaques on lawns using *S. ruminicola* KCTC 43306 strain from the Korean Collection for Type Culture (KCTC) as the host. Transmission electron microscopy (TEM) analysis revealed that phages vB_SbRt-pBovineB21 and vB_SbRt-pBovineS21 have an icosahedral head of 49.2 and 46.2 nm in diameter, respectively. They also exhibited short non-contractile tails of 13.3 and 12.5 nm, respectively (Fig. 1). Accordingly, both phages were morphologically classified as members of the *Podoviridae* family. The host range analysis of isolated phages revealed that among the *S. equinus* strains, lysis was lower for isolates from *Bos taurus* (15.4%) and *Bos taurus coreanae* (35.3%). However, most isolates from *Capra aegagrus hircus*, including *S. ruminicola* (100%), *S. equinus* (64.3–71.4%), and *S. lutetiensis* (100%) were lysed by these phages (Table 1). No lysis was observed in the nine type strains of SBSEC and *S. agalactiae*. Notably, both phages could infect all type strains of the other genera, *Lactobacillus* spp. and *Lactococcus lactis* subsp. *lactis*, used in this study.

**Table 1:** Host range of the isolated SBSEC phages used in this study.

Bacterial strain	Deposit no	Isolate source	SBSEC phages	
			vB_SbRt-pBovineB21	vB_SbRt-pBovineS21
*Streptococcus ruminicola*				
CNU_77-61 (Host)	KCTC 43306	*Capra aegagrus hircus*	+	+
CNU_G2	KCTC 43308^ T^	*Bos taurus*	–	–
CNU_G3	KCTC 43307	*Bos taurus*	–	–
*Streptococcus equinus*				
CNU_5	KCCM 90360	*Bos taurus*	–	–
CNU_77-2	KCCM 90361	*Bos taurus*	–	–
CNU_77-3	KCCM 90362	*Bos taurus*	–	–
CNU_11	KCCM 90363	*Bos taurus*	–	–
CNU_77-16	KCCM 90364	*Bos taurus*	+	+
CNU_77-20	KCCM 90365	*Bos taurus*	–	–
CNU_77-23	KCCM 90366	*Bos taurus*	–	–
CNU_77-27	KCCM 90367	*Bos taurus*	+	+
CNU_GF	KCCM 90354	*Bos taurus*	–	–
CNU_G1	KCCM 90381	*Bos taurus*	–	–
CNU_G4	KCCM 90357	*Bos taurus*	–	–
CNU_G5	KCCM 90358	*Bos taurus*	–	–
CNU_G6	KCCM 90359	*Bos taurus*	–	–
CNU_9	KCCM 90368	*Bos taurus coreanae*	–	–
CNU_77-8	KCCM 90369	*Bos taurus coreanae*	–	+
CNU_77-11	KCCM 90370	*Bos taurus coreanae*	+	+
CNU_77-12	KCCM 90371	*Bos taurus coreanae*	+	+
CNU_77-14	KCCM 90372	*Bos taurus coreanae*	+	+
CNU_77-29	KCCM 90373	*Bos taurus coreanae*	–	–
CNU_15	KCCM 90374	*Bos taurus coreanae*	–	–
CNU_20	KCCM 90375	*Bos taurus coreanae*	–	–
CNU_21	KCCM 90376	*Bos taurus coreanae*	+	+
CNU_77-35	KCCM 90377	*Bos taurus coreanae*	–	–
CNU_77-37	KCCM 90378	*Bos taurus coreanae*	–	–
CNU_77-40	KCCM 90379	*Bos taurus coreanae*	–	–
CNU_25	KCCM 90380	*Bos taurus coreanae*	–	–
CNU_27	KCCM 90381	*Bos taurus coreanae*	+	+
CNU_77-43	KCCM 90382	*Bos taurus coreanae*	+	
CNU_77-47	KCCM 90383	*Bos taurus coreanae*	–	–
CNU_77-50	KCCM 90384	*Bos taurus coreanae*	–	–
CNU_29	KCCM 90385	*Bos taurus coreanae*	+	+
CNU_30	KCCM 90386	*Bos taurus coreanae*	+	+
CNU_32	KCCM 90387	*Bos taurus coreanae*	–	–
CNU_77-51	KCCM 90388	*Bos taurus coreanae*	+	+
CNU_77-55	KCCM 90389	*Bos taurus coreanae*	+	+
CNU_77-56	KCCM 90390	*Bos taurus coreanae*	–	–
CNU_77-57	KCCM 90391	*Bos taurus coreanae*	–	+
CNU_77-60	KCCM 90394	*Bos taurus coreanae*	–	–
CNU_77-68	KCCM 90398	*Bos taurus coreanae*	–	–
CNU_41	KCCM 90399	*Capra aegagrus hircus*	+	+
CNU_42	KCCM 90400	*Capra aegagrus hircus*	+	+
CNU_77-72	KCCM 90401	*Capra aegagrus hircus*	+	+
CNU_77-77	KCCM 90403	*Capra aegagrus hircus*	+	+
CNU_77-78	KCCM 90404	*Capra aegagrus hircus*	+	+
*Streptococcus lutetiensis*				
CNU_33	KCCM 90393	*Capra aegagrus hircus*	+	+
CNU_77-62	KCCM 90396	*Capra aegagrus hircus*	+	+
CNU_77-64	KCCM 90397	*Capra aegagrus hircus*	+	+
CNU_77-76	KCCM 90402	*Capra aegagrus hircus*	+	+
Type strains of SBSEC				
* S. equinus*	ATCC 9812^ T^	*Equus ferus caballus*	–	–
* S. bovis*	ATCC 33317^ T^	*Bos taurus*	–	–
* S. infantarius* subsp. *infantarius*	ATCC BAA-102^ T^	*Homo sapiens*	–	–
* S. gallolyticus* subsp. *gallolyticus*	DSM 16831^ T^	*Phascolarctos cinereus*	–	–
* S. gallolyticus* subsp. *pastuerianus*	NEM 1202^ T^	*Homo sapiens*	–	–
* S. gallolyticus* subsp. *macedonicus*	DSM 15879^ T^	Cheese	–	–
* S. lutetiensis*	NCTC 13774^ T^	*Homo sapiens*	–	–
* S. alactolyticus*	ATCC 43077^ T^	*Sus domesticus*	–	–
* S. agalactiae*	KCCM 11957^ T^	*Homo sapiens*	–	–
*Lactobacillus* (*L.*) spp.				
* L. plantarum* subsp. *plantarum*	KCTC 3108^ T^	Cabbage	+	+
* L. casei*	KCCM 12452^ T^	Cheese	+	+
* L. sakei*	KCCM 40264^ T^	Yeast starter	+	+
*Lactococcus lactis* subsp.* lactis*	KCCM 41572^ T^	Unknown	+	+
Infection rate			26/64 (40.6%)	27/64 (42.2%)

### Adsorption rate and one-step growth analysis

**Figure 2:**
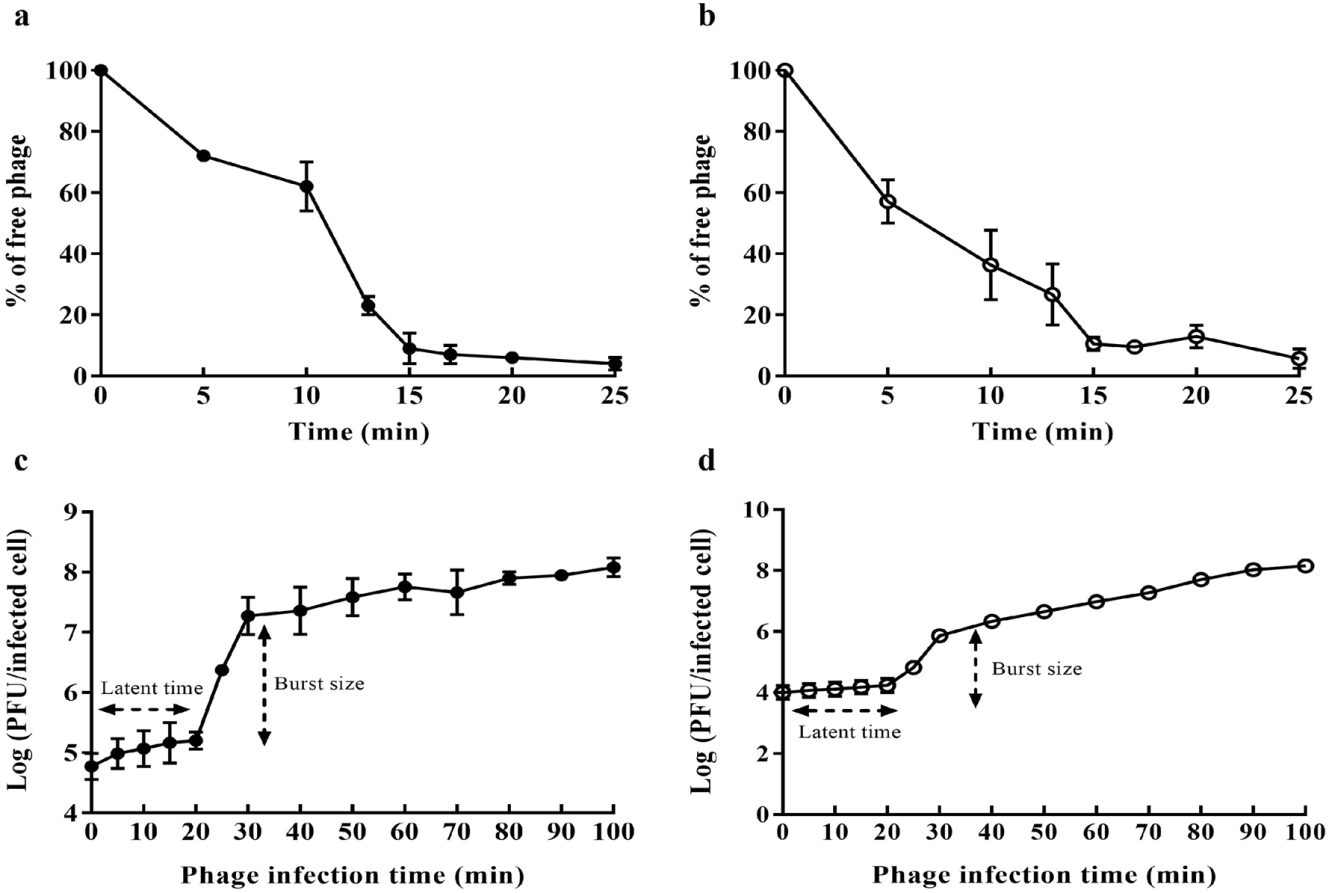
Adsorption rate and one-step growth curve of the isolated SBSEC phages. The adsorption rates of phages vB_SbRt-pBovineB21 (**a**) and vB_SbRt-pBovineS21 (**b**) decreased to less than 80% at 15 min. One-step growth curves of phages vB_SbRt-pBovineB21 (**c**) and vB_SbRt-pBovineS21 (**d**) show a latent time of 20 min and burst size of 367.5 and 198.3 PFU/mL, respectively. In all experiments, a triplicate assay was independently undertaken using *S. ruminicola* KCTC 43,306. The error bars indicate the standard error of the mean.

Over 80% of the free SBSEC phages were decreased within approximately 15 min, indicating that the phages were adsorbed efficiently on the host strain, (Fig. 2a,b). The latent time and burst size were determined using the one-step growth curve of the isolated phages against *S. ruminicola* KCTC 43306 at the optimal multiplicity of infection (MOI) of 0.0001. The latent time and burst size of phage vB_SbRt-pBovineB21 were approximately 20 min and 367.5 PFU/infected cells, respectively, as shown in Fig. 2c. Furthermore, the phage vB_SbRt-pBovineS21 had a latent time and burst size of approximately 20 min and 198.3 PFU/infected cells, respectively (Fig. 2d).

### Thermal and pH stability

**Figure 3:**
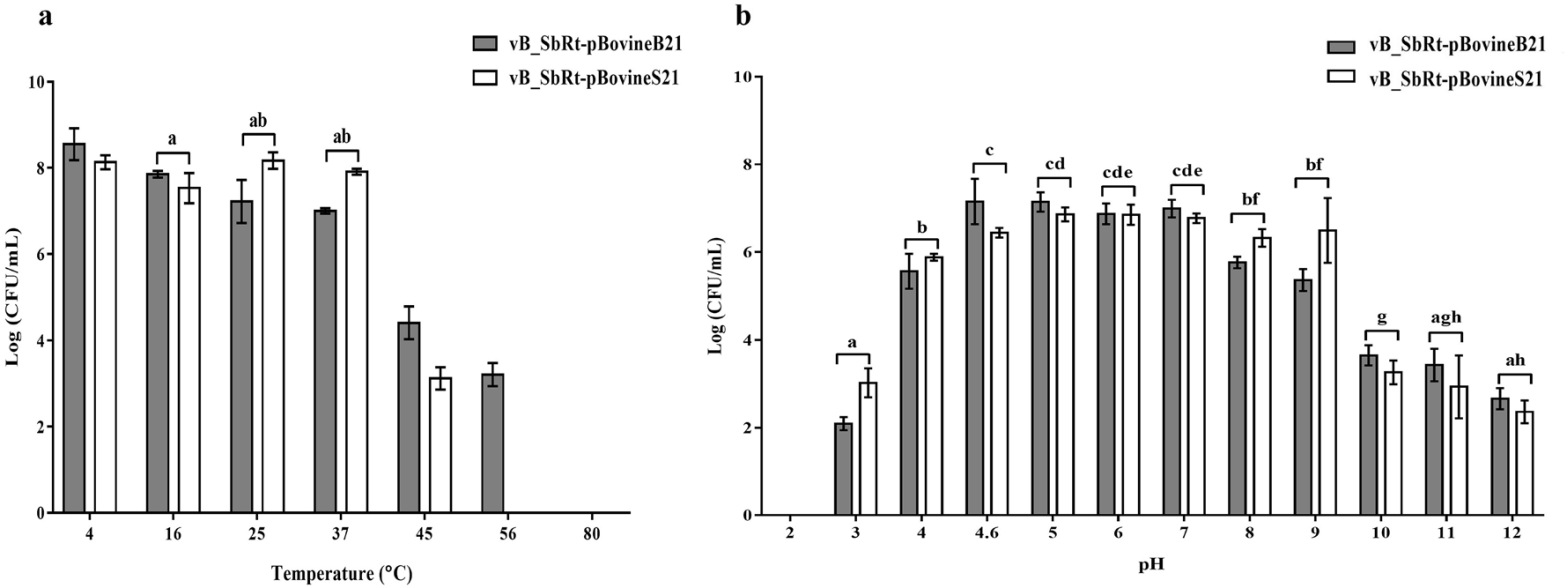
Stability of the isolated SBSEC phages under different thermal and pH conditions. (**a**) The thermal stability of both isolated phages at 4, 16, 25, 37, 45, 56, and 80 °C. (**b**) The pH stability of the isolated phages at pH 2, 3, 4, 4.6, 5, 6, 7, 8, 9, 10, 11, and 12. All experiments were conducted independently in triplicate. The vertical lines indicate the standard error of the mean. The bars marked with the same letters indicate no significant difference (p < 0.05).

The stability of the isolated SBSEC phages was determined at various temperatures and pH values. Regarding thermal stability results, the phage vB_SbRt-pBovineB21 was relatively stable at 4, 16, 25, 37, 45, and 56 °C for 3 h, but was completely inactivated after incubation at 80 °C for 3 h. The phage vB_SbRt-pBovineS21 was relatively stable at 4, 16, 25, 37, and 45 °C, but was completely inactivated after incubation at 56 and 80 °C for 3 h (Fig. 3a) The pH stability results revealed that both phages survived a pH range of 3–12 and were completely inactivated at pH 2, thus suggesting that no phage could survive the strongly acidic and alkaline environments (Fig. 3b).

### Bacteriolytic activity

**Figure 4:**
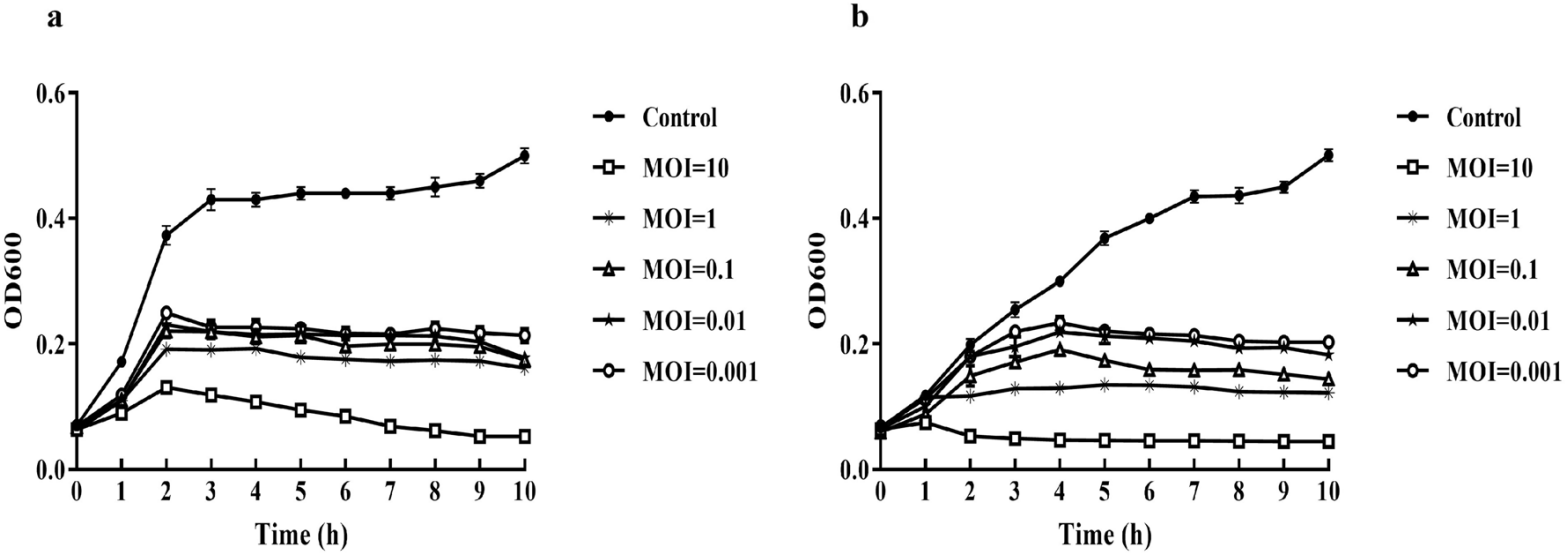
The bacteriolytic effects of the isolated SBSEC phages at the different MOIs. Cell lysis activities of phages vB_SbRt-pBovineB21 (**a**) and vB_SbRt-pBovineS21 (**b**) at an MOIs of 10, 1, 0.1, 0.01, and 0.001 against *S. ruminicola* KCTC 43,306 and without phage used as control. Each experiment was conducted independently in triplicate. The error bars indicate the standard error of the mean.

The bacteriolytic effects of the isolated SBSEC phages against *S. ruminicola* KCTC 43306 were compared at different MOIs. As presented in Fig. 4, the optical density (OD; 600 nm) values of all MOIs (0.001, 0.01, 0.1, 1, and 10) rapidly increased during the first 2 h. Subsequently, the OD 600 nm values of phage-treated groups were considerably lower than those of the positive control (MOI of 0). In both phages, the OD 600 nm values of phage-treated groups were sustained (MOIs of 0.001, 0.01, 0.1, and 1) or constantly decreased (MOI of 10) up to 10 h after phage inoculation, thus indicating that the growth of the host cells was effectively inhibited by the phage.

### Genomic features of the isolated SBSEC phages

**Figure 5:**
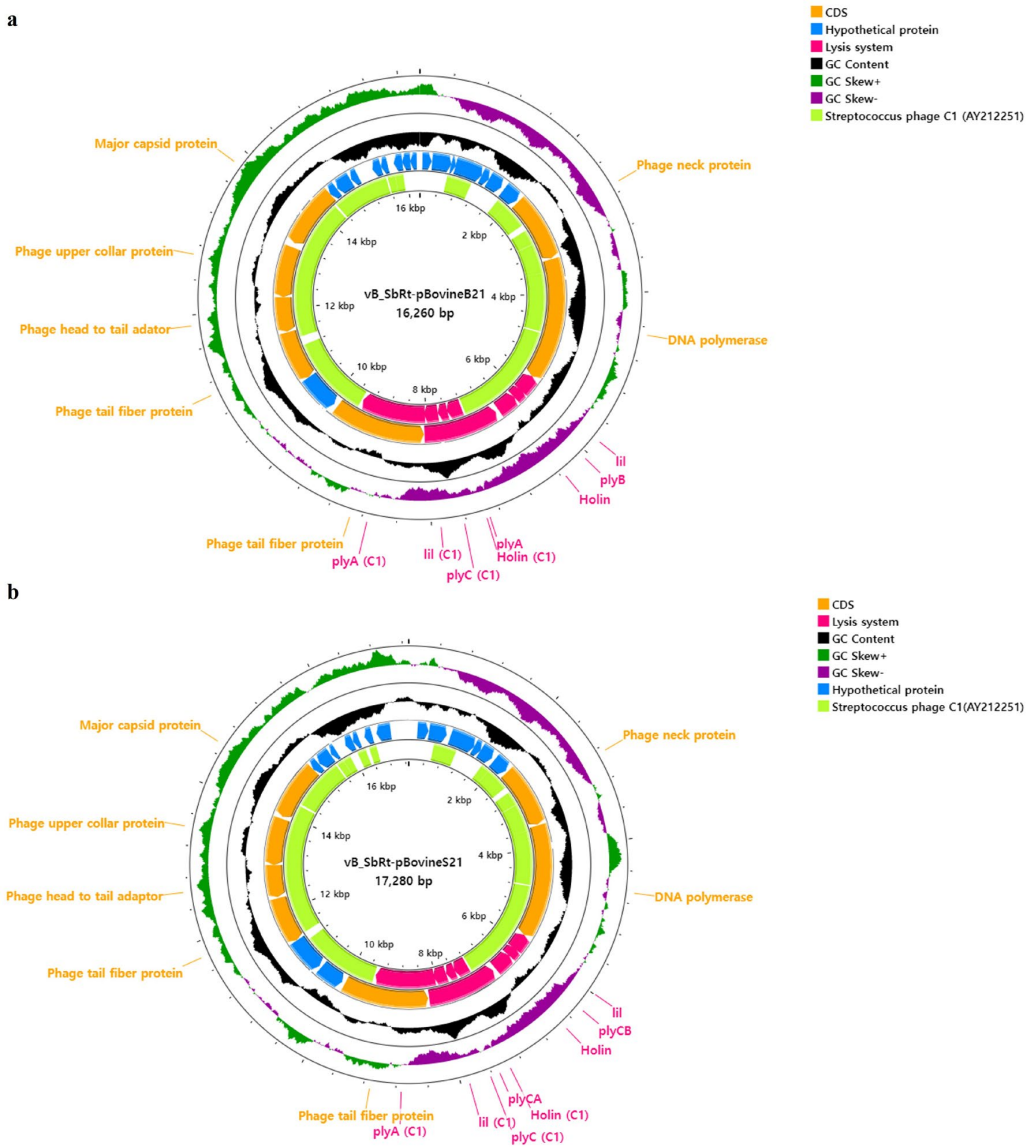
Genome maps of the isolated SBSEC phages. The circular genome maps of phages vB_SbRt-pBovineB21 (**a**) and vB_SbRt-pBovineS21 (**b**) indicate each ORF predicted in colored based on the functional categories: hypothetical protein (blue), lysis system (pink), and additional phage-associated protein (orange). The inner lane, yellow-green, represents blast homology for genomes of *Streptococcus* phage C1 (AY212251.1). GC content, positive GC Skew, and negative GC skew are indicated as black, green, and purple, respectively.

**Figure 6:**
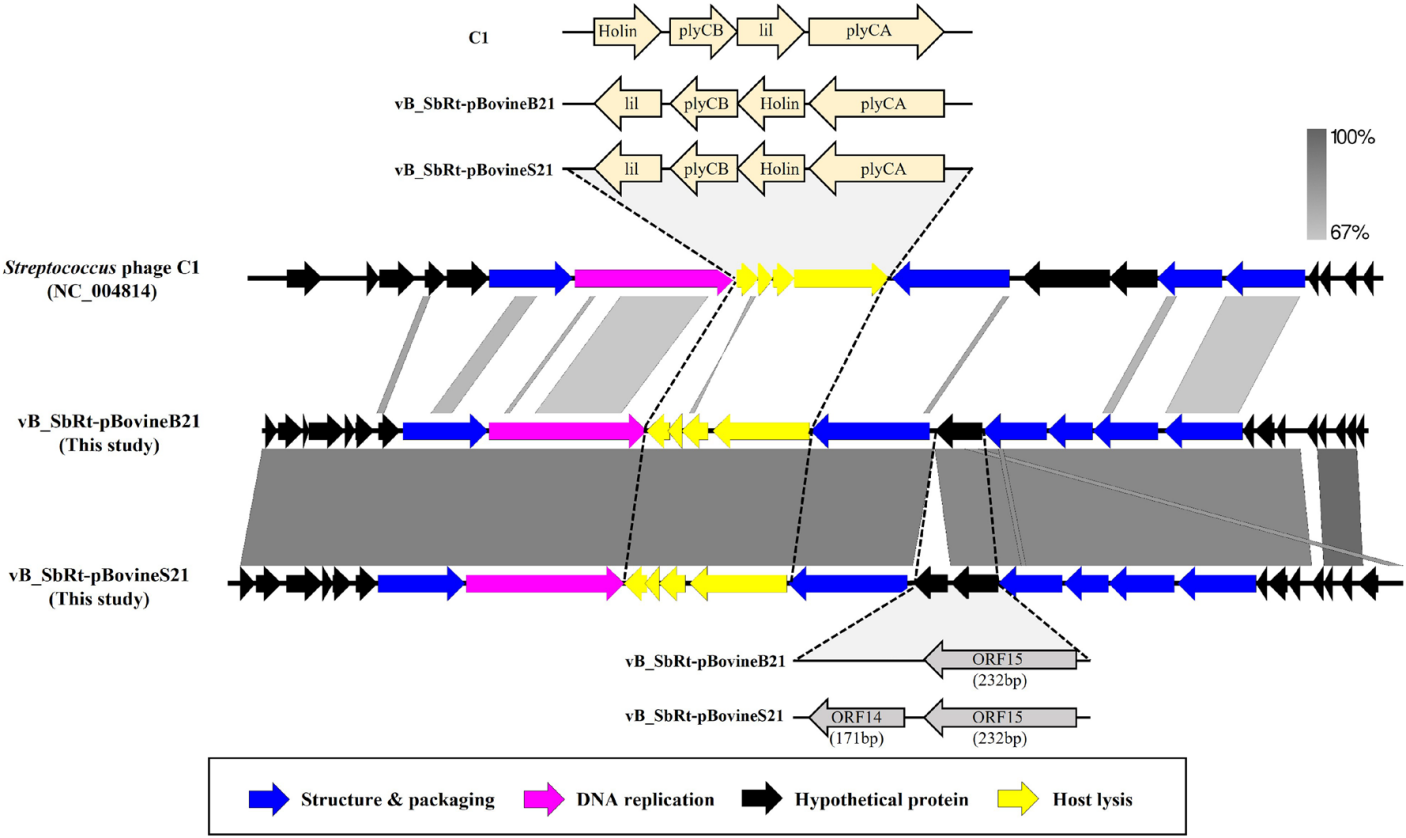
Genomic comparisons of the isolated SBSEC phages and *Streptococcus* phage C1. The linear visualization is represented by coding regions of phage genomes with colored arrows. Functionally annotated ORFs in relation to phage-associated proteins, such as structure and packaging, DNA replication, host lysis, and hypothetical protein were colored blue, pink, yellow, and black. Additionally, the sequence similarity is indicated by the gray intensity.

The genome of phages vB_SbRt-pBovineB21 and vB_SbRt-pBovineS21 comprised of a linear double-stranded DNA of 16,260 bp and 17,280 bp in length with a G + C content of > 33% and predicted 27 and 26 open reading frames (ORFs), respectively. The genome maps are shown in Fig. 5a and Fig. 5b. In both phages, 10 ORFs were predicted as functional proteins, including encapsidation protein, DNA polymerase, putative lysis system-associated proteins, phage tail protein, tail fiber protein, head-to-tail adapter, upper collar protein, and major capsid protein. Additionally, 13 conserved domains were detected in functionally predicted ORFs based on homology comparisons of phage-originated proteins. Over 10 ORFs in phages vB_SbRt-pBovineB21 and vB_SbRt-pBovineS21 were similar (amino acid identities; 25.5–63.6%) to the predicted proteins from *Streptococcus* phage C1^19^. In addition, over four ORFs were similar (amino acid identities < 45%) to other *Podoviridae* phages available in the GenBank database, indicating that isolated SBSEC phages possess unique genomic structures. The remaining ORFs were predicted as hypothetical proteins, with unknown proteins annotated using BLASTP searches. Additionally, transmembrane domains were predicted in three ORFs and no signal peptide was predicted in all the ORFs of both phages (Supplementary Tables 1 and 2). tRNA genes and bacterial virulence- and antimicrobial resistance-associated genes were not detected in the genomes of both the isolated phages. The PhageTerm analysis was predicted to be permuted with redundant ends in the isolated phages. Particularly, the genomes of phages vB_SbRt-pBovineB21 and vB_SbRt-pBovineS21 had high similarities to 18 predicted ORFs, and 13 putative functional proteins divided into four functional groups: two nucleotide metabolisms, four viral structure and packaging, four lysis, and three tail structures. However, both phages distinctly differed in the genes present in the intergenic regions between ORF 13 and ORF 15. In phage vB_SbRt-pBovineS21, ORF 14 (171 bp) and ORF 15 (232 bp) were located next to ORF 13, encoding a tail protein of 583 bp in a row; however, the ORF 14 presented in phage vB_SbRt-pBovineS21 was not detected in phage vB_SbRt-pBovineB21 (Fig. 6).

### Genomic comparison and phylogenetic analysis of the isolated SBSEC phages

**Figure 7:**
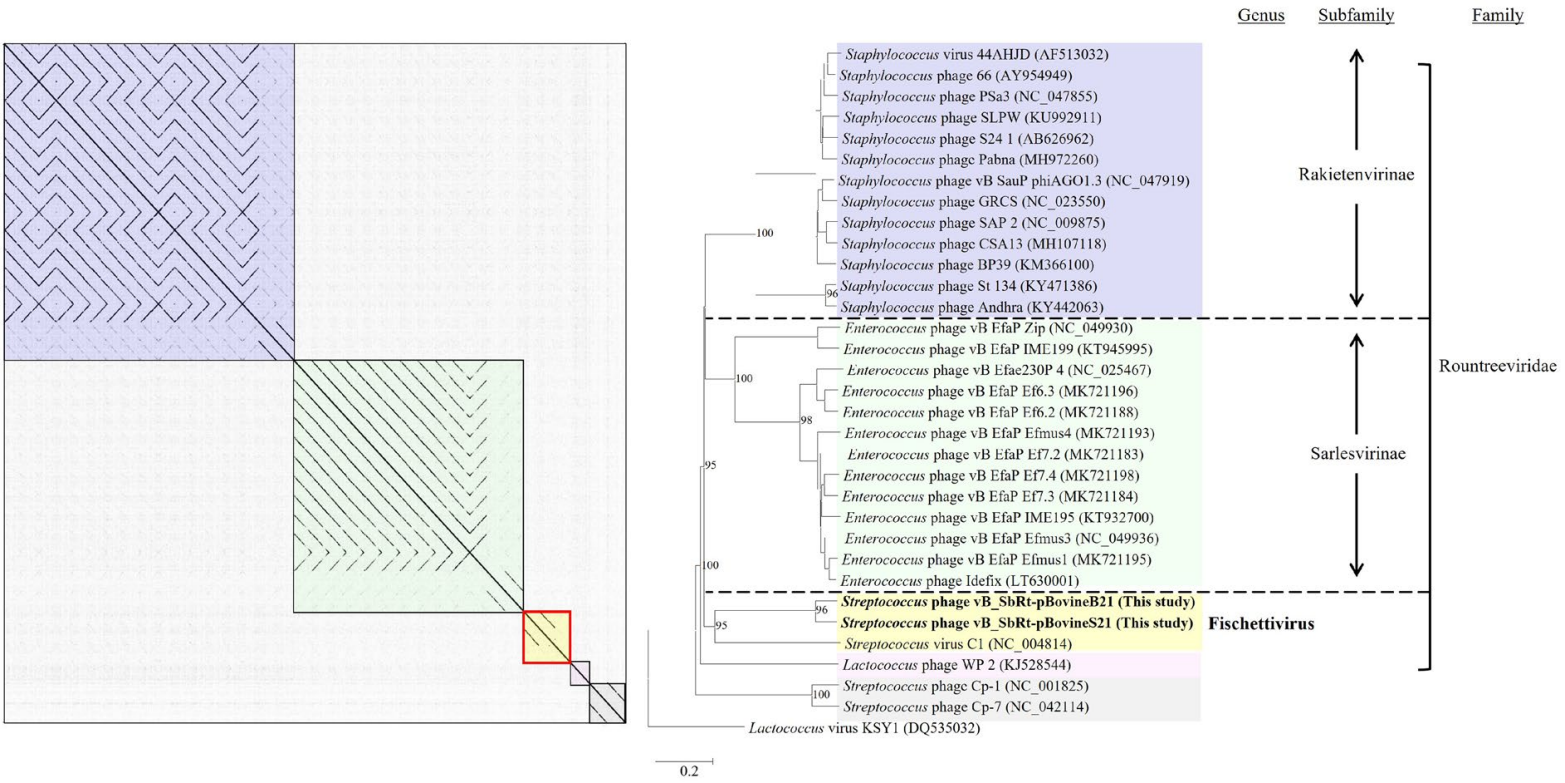
Phylogenetic analysis of the isolated SBSEC phages based on the sequenced complete genome. The phylogenetic tree and dot plot indicate that both phages cluster with *Streptococcus* phage C1 as new members of the *Fischettivirus* genus. The colored boxes are represented by members of the *Rakietenvirinae* subfamily (purple), *Sarlesvirinae* subfamily (green), *Fischettivirus* genus (yellow), and *Negarvirus* genus (pink), respectively, belonging to the *Rountreeviridae* family. The phages used in this study are highlighted in bold. The outgroup is the *Lactobacillus* phage KSY1 (DQ535032.1).

**Figure 8:**
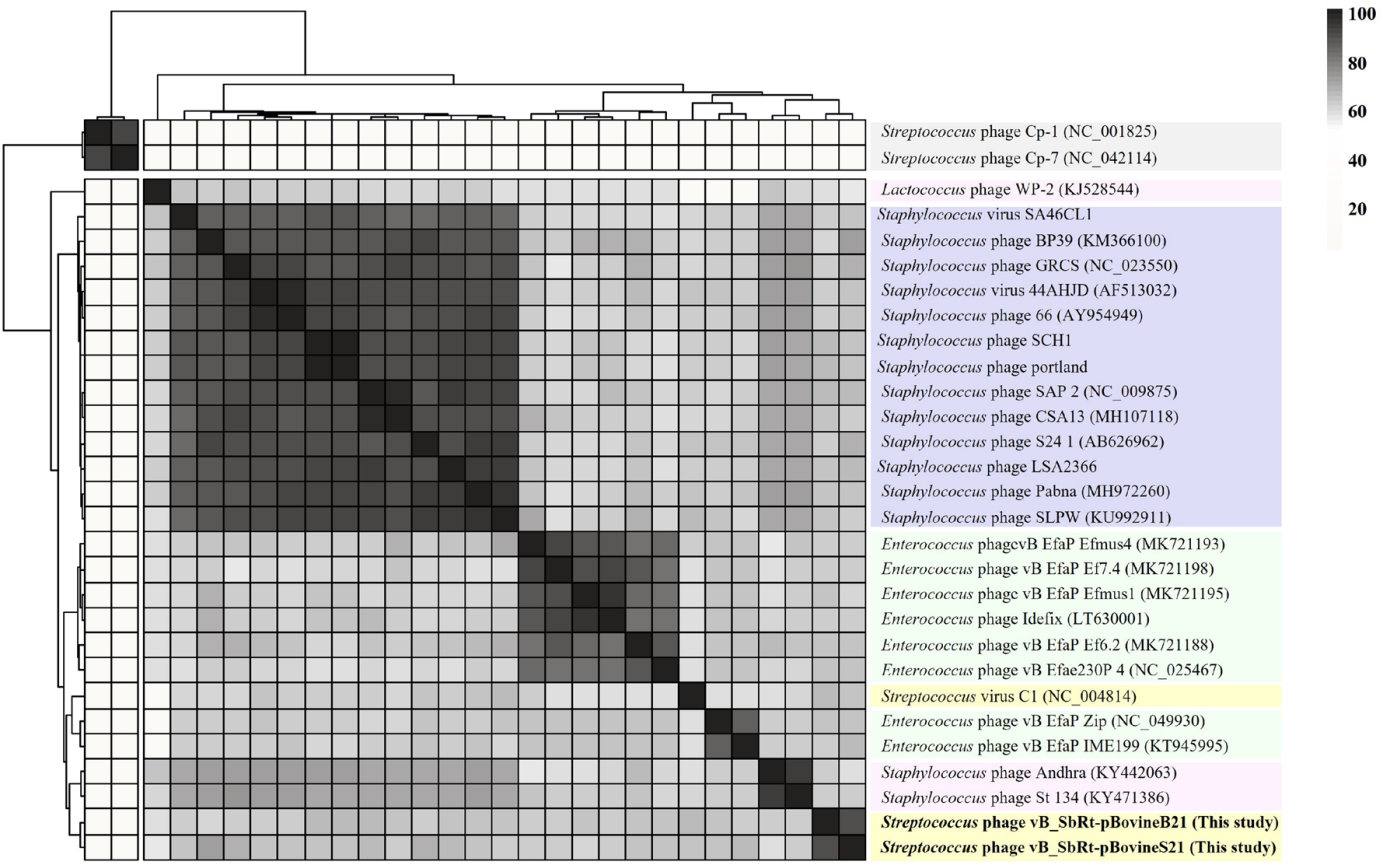
Heat map of the isolated SBSEC phages isolated in this study. The map drawn using the ANI values of 29 genomes, including the isolated SBSEC and *Rountreeviridae* phages, demonstrates the clustering based on intergenomic similarity. The phages used in this study are indicated in bold.

Comparison of the sequenced genome of phages vB_SbRt-pBovineB21 and vB_SbRt-pBovineS21 to other phages currently available in the GenBank database suggested that the isolated phages were most similar to *Streptococcus* phage C1 (AY212251.1) with < 77% identity based on the < 2% query coverage (Supplementary Table 3). Therefore, based on the classification standards proposed by the ICTV^20^, the isolated phages were classified into the *Rountreeviridae* family (Supplementary Table 4). Additionally, based on the similarity with the amino acid sequence, comparative genomic analysis was performed between the isolated SBSEC phages and phage C1 using Easyfig. The analysis revealed that the overall genomic contents and arrangements of the SBSEC phages were similar to those of phage C1, the sole member of the *Fischettivirus* genus in the *Rountreeviridae* family. However, the SBSEC phages showed several differences from the phage C1: (i) Although most of the genes with similar functions were sequentially located in the genome of three phages, the ranges of the amino acid identity of the predicted ORFs in the SBSEC phages against phage C1 were less than 63.6%. (ii) Although the SBSEC phages possessed a similar host lysis system (*holin*-*plyCB*-*lil*-*plyCA*) to the phage C1^19^, the four genes (*lil* (ORF 10 and ORF 9), *plyCB* (ORF 11 and ORF 10), *holin* (ORF 12 and ORF 11), and *plyCA* (ORF 13 and ORF 12)) were found inversely inserted in the sequenced genomes and the internal locations of *holin* and *lil* were changed around *plyCB* (Fig. 6) Thus, genomic evidence strongly supports that vB_SbRt-pBovineB21 and vB_SbRt-pBovineS21 phages are distinctly different from the phage C1 and, are therefore, considered as new species. To determine the taxonomic position of the SBSEC phages vB_SbRt-pBovineB21 and vB_SbRt-pBovineS21, phylogenetic analyses and dot plot comparisons were conducted using the complete genome sequences of the two isolated phages and 30 other phages belonging to the *Rountreeviridae* family (Supplementary Table 4). Although the two SBSEC phages were clustered together with *Streptococcus* phage C1, phages vB_SbRt-pBovineB21 and vB_SbRt-pBovineS21 were separated into different sub-branches as shown in Fig. 7. Additional phylogenetic analyses using functional proteins, including the major capsid protein (Supplementary Fig. 1a) and DNA polymerase (Supplementary Fig. 1b) also revealed consistent results with the genome-based cladistic analysis. Moreover, the estimated average nucleotide identity using OrthoANI between phages vB_SbRt-pBovineB21 and vB_SbRt-pBovineS21 was 89.8%, and the resulting heat map suggested no clustering for the two SBSEC phages and *Streptococcus* phage C1 (Fig. 8). Based on these results, the newly isolated SBSEC phages could be considered as new species of the *Fischettivirus* genus.

### Anti-biofilm activity of the isolated SBSEC phages

**Figure 9:**
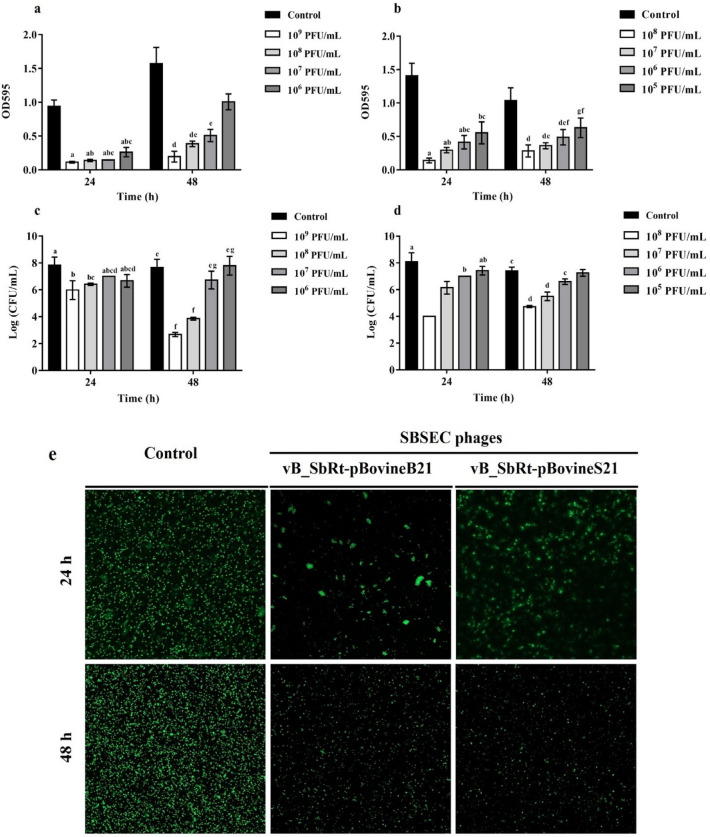
Biofilm prevention ability of the isolated SBSEC phages. The effects of phages vB_SbRt-pBovineB21 (**a**) and vB_SbRt-pBovineS21 (**b**) on total biomass of biofilm formation at different concentrations for 24 and 48 h. The effects of phages vB_SbRt-pBovineB21 (**c**) and vB_SbRt-pBovineS21 (**d**) on viable bacterial cells of biofilms at different concentrations for 24 and 48 h. Control was a bacteria culture without phage. Each experiment was independently conducted in triplicate using *S. ruminicola* KCTC 43,306. The values marked by the different letters indicate significant differences (p < 0.05). (**e**) CLSM images of *S. ruminicola* KCTC 43,306 biofilm treated with two isolated phages (10^7^ PFU/mL) for 24 and 48 h. Green fluorescence observed using Syto 9 in the images indicates live cells. The scale bar is 100 μM.

**Figure 10:**
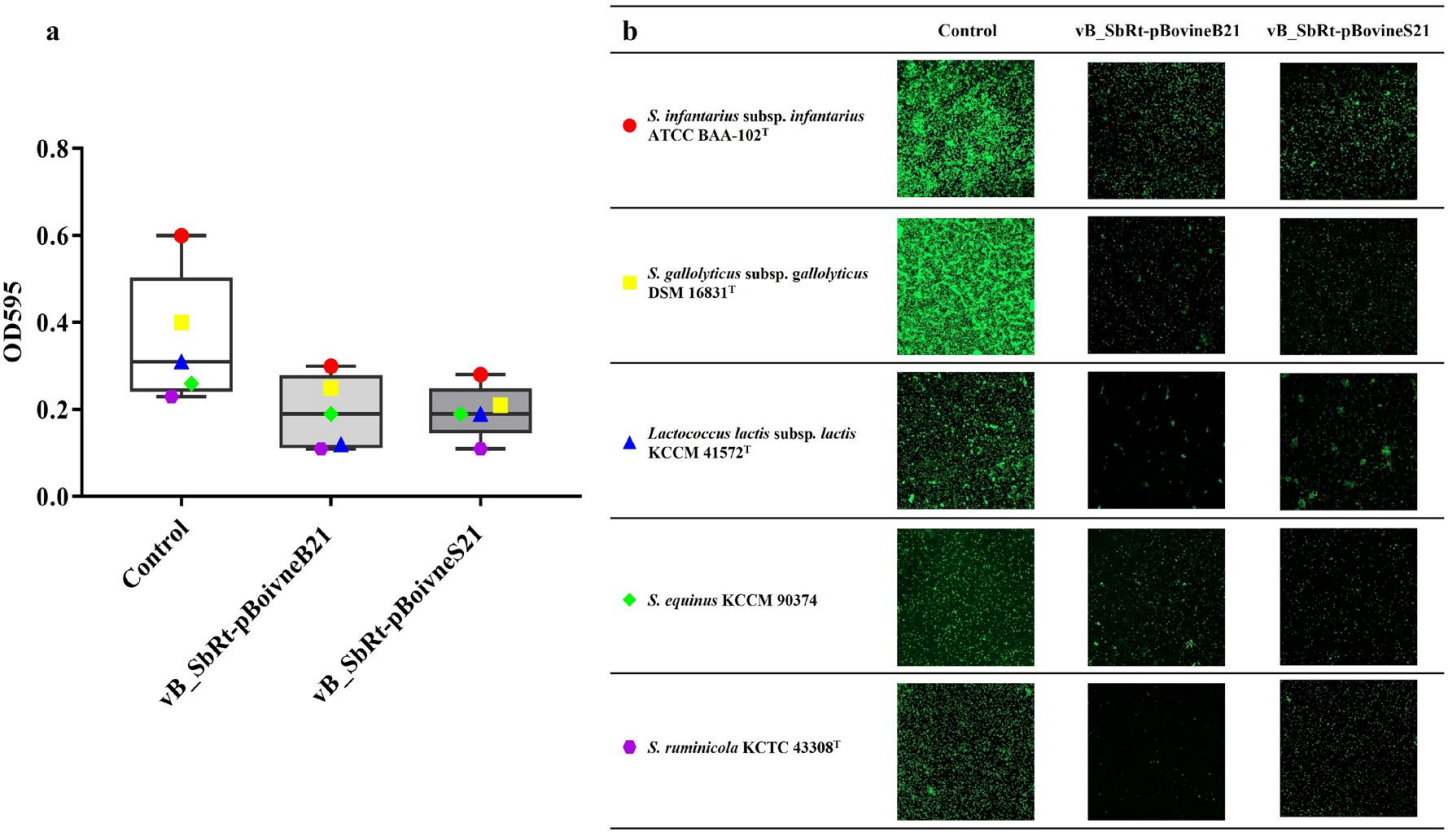
Biofilm prevention ability of the isolated SBSEC phages against the five bacterial strains used in this study. (**a**) The effects of phages vB_SbRt-pBovineB21 and vB_SbRt-pBovineS21 on total biomass of biofilm formation at 10^7^ PFU/mL for 24 h. Control was a bacteria culture without phage. The values plotted in the box and whiskers graph using GraphPad Prism 7 revealed all points along with the minimum and maximum. Each experiment was independently undertaken in triplicate. (**b**) CLSM images of selected-five bacterial strains biofilm treated with the two isolated phages (10^7^ PFU/mL) for 24 h. Green fluorescence observed using Syto 9 indicate live cells. The scale bar is 100 μM.

To evaluate the effect of phages on SBSEC biofilm formation, *S. ruminicola* KCTC 43,306 was incubated with different titers of phage suspension. The results of the biofilm prevention assay indicated that both phages at different concentrations (10^9^, 10^8^, 10^7^, 10^6^, and 10^5^ PFU/mL) steadily reduced the total biomass than that in the control group (Fig. 9a and 9b). In vB_SbRt-pBovineB21, the host cell viability was reduced by 5 logs CFU/mL in the biofilm formed after 24 h incubation, reaching a maximum of 2 log CFU/mL reduction after 48 h (Fig. 9c). In vB_SbRt-pBovineS21, a gradual reduction of biofilm formation was observed. The viable cells decreased by 4 logs CFU/mL when compared to the control after 24 and 48 h incubation (Fig. 9d). The lytic effects of phages on biofilms and the presence of viable cells were also analyzed using a confocal laser scanning microscope (CLSM) and the phages showed the potential to control the biofilm formed by *S. ruminicola* (Fig. 9e). The potential biofilm prevention ability of the phages was also assessed using a total of five other phage-susceptible strains (*S*. *infantarius* subsp. *infantarius* ATCC BAA-102^ T^, *S. gallolyticus* subsp. *gallolyticus* DSM 16831^ T^, *S. equinus* KCCM 90,374, *S*. *ruminicola* KCTC 43308^ T^, and *L. lactis* subsp. *lactis* KCCM 41572^ T^) which showed relatively higher biofilm production (data not shown). The results indicated that phages vB_SbRt-pBovineB21 and vB_SbRt-pBovineS21 were highly efficient in preventing biofilm formation of the five-selected strains with a relatively low dose of phage concentration (10^7^ PFU/ml) for 24 h (Fig. 10).

## Discussion

SBSEC is a commensal bacterium that inhabits the gastrointestinal tract of animals and humans; however, its proliferation acts as a trigger for lactic subacute acidosis in ruminants. Moreover, several representative species known to be pathogenic in patients with infectious diseases have also been reported. Recently, a new member of SBSEC, *S. ruminicola*, was proposed by our group^3^. Nevertheless, little is known about the biodiversity of SBSEC and the phages infecting this bacterial complex. This is the first study to report the biological and genetic properties of both lytic phages infecting representative SBSEC, thereby leading to the potential control of ruminal microbiota (Table 2). The isolated phages have largely infected the SBSEC of both type strains and wild isolates from the rumen^21^. Interestingly, the isolated phages showed broad lytic activity against other lactic acid-producing bacteria, such as *Lactobacillus* spp.^22,23^ and *Lactococcus* sp.^24^, which rapidly produces lactic acid in the rumen^25^. This result suggests that the newly isolated phages may have the potential to prevent excessive accumulation of lactate produced by predominant rumen bacteria during metabolic disorders.

**Table 2:** General characteristics of the isolated SBSEC phages vB_SbRt-pBovineB21 and vB_SbRt-pBovineS21.

Characteristics	SBSEC phages	
	vB_SbRt-pBovineB21	vB_SbRt-pBovineS21
Isolate source	Feces	Sewage
Morphotype		
Plaque appearance	Cloudy	Cloudy
Head		
Structure	Icosahedral capsid	Icosahedral capsid
Length (nm)	49.2	46.2
Tail		
Structure	Short non-contractile	Short non-contractile
Length (nm)	13.3	12.5
Similar to family	*Podoviridae*	*Podoviridae*
Biotype		
Time for maximum adsorption (min)	15	15
Latent time (min)	20	20
Burst size (PFU/infected cell)	367.5	198.3
Optimal temperature range	4–37	4–37
Optimal pH range	5–9	5–9
Genotype		
Size (bp)	16,260	17,280
G + C (%)	33.8	33.5
Contig	1	1
Total genes	27	27
Protein-coding genes (CDS)	27	27
tRNAs	0	0
VFG*	0	0
ARG**	0	0
Similar to family	*Rountreeviridae*	*Rountreeviridae*

*VF; Virulence Factors were identified using the VFDB (http://www.mgc.ac.cn/VFs/).

**ARG; Antibiotic Resistance Genes were identified using the ARG-ANNOT (Antibiotic Resistance Gene-ANNOTation).

The morphological features of the isolated SBSEC phages were similar to those of *Streptococcus* phage C1^19^, which formerly belonged to the *Podoviridae* family and is currently a member of *Rountreeviridae*, according to the taxonomy of the ICTV^20^. Moreover, the lytic characteristics of the isolated SBSEC phages exhibited efficient antibacterial activity against SBSEC, after both the phages adsorbed the host bacteria within 15 min. The latent time, defined as the time between adsorption and bacterial cell lysis onset, was 20 min. In addition, the burst size, defined as the number of phages released by the infected host cell, was 198–367.5 PFU per infected cell. These findings indicated that the isolated phages displayed a rapid adsorption, a shorter latent period, and a significantly larger burst size than that of *Enterococcus* phage vB_EfaP_IME195 with 30 min and approximately 120 PFU/cell^26^, *Staphylococcus* phage CSA13 with 20 min and approximately 230 PFU/mL^27^ in the *Rountreeviridae* family, and *Streptococcus* phage Cp-1 with 50 min and approximately 9 PFU/mL^28^ in the *Salasmaviridae* family.

The stability of the isolated phages under various (including harsh) environmental conditions, such as temperature and pH, is a significant property for using them as potential biocontrol agents. Both the isolated SBSEC phages were relatively stable between 4–45 °C, suggesting their adaptation to metabolic activity in the rumen environment at approximately 38°C^29^. Furthermore, both phages were stable at a pH between 3–12 , indicating strong pH tolerance for extremely acidic and alkaline environments. The pH conditions for the optimal activity of starch-degrading bacteria in the rumen ranges between 5.4–6^30^, and ruminal subacute acidosis conditions should be maintained between pH 5.2–6^31^. These findings are significant, owing to the ability of the isolated phages to survive at low pH, which may prevent ruminal acidosis caused by lactic acid-producing bacteria, including SBSEC, in the ruminants. Moreover, the environmental stability of the isolated phages was consistent with that of the *Escherichia coli* phage isolated from cattle feces, which reported an optimal tolerance between 37–40 °C and pH 6.3–8, respectively^32^. The bacteriolytic effects were shown as 2 log CFU/mL reduction within 2 h at the lowest MOI of 0.001 when compared to the control group. This effectively aggravates the host bacteria survival at very low concentrations and is advantageous for potential applications.

The genomic characteristics of both SBSEC phages were most similar to those of *Streptococcus* phage C1^19^. However, the newly isolated phages showed several different genomic characteristics when compared to phage C1 and can be classified as new members in the genus *Fischettivirus* as follows: First, the genome of both SBSEC phages overall demonstrated a relatively low identity (< 77%) at the nucleotide level with 2% coverage and distinct genomic arrangements when compared to the phage C1. Second, only half of all predicted ORFs in both SBSEC phages were annotated as hypothetical proteins. Each of the three proteins (ORF 7, ORF 11, and ORF 20 in phage vB_SbRt-pBovineB21 and ORF 6, ORF 10, and ORF 20 in phage vB_SbRt-pBovineS21) was predicted to encode phage-associated genes. Third, both the SBSEC phages possessed a similar but distinct bacterial lysis systems from phage C1. *Streptococcus* phage C1 has been reported to possess a novel lysin, PlyC, composed of two specific structures identified as *plyCA* and *plyCB*, which encode catalytic and cell wall-binding domains, respectively^19,33,34^. The sequenced genomes of the newly isolated phages in this study also encoded two putative proteins each (ORF 11 and ORF 13 in vB_SbRt-pBovineB21; ORF 10 and ORF 12 in vB_SbRt-pBovineS21) with conserved domains, predicted to be *plyCA* and *plyCB*. Nonetheless, the predicted genes displayed low homology and arrangement distinction with phage C1 (Fig. 6). These results revealed that the newly isolated SBSEC phages have lysis systems different from that of phage C1. Thus, additional studies on the detailed characteristics of the potential activity of lysin-associated enzymes derived from the isolated phages are required to confirm their uniqueness in our future analysis.

To date, little is known concerning the presence and exact role of biofilms caused by SBSEC. Several strains of *S. galloyticus*, including three subspecies known as causative pathogens of infective endocarditis, have higher biofilm formation ability and are significantly associated with antibiotic resistance^35,36^. However, the biofilm-producing abilities of some strains of *S*. *lutetiensis* and *S*. *infantarius* subsp. *infantarius* isolated from dairy products could be regarded as an advantageous characteristic^36^. Similar to the aforementioned SBSEC strains, the host strain KCTC 43,306 could produce biofilm and we were able to confirm the presence of anti-biofilm activity in the isolated phages which can efficiently reduce biofilm formation and the viable bacterial cells in the biofilms. Interestingly, the formation of biofilms of five strains of SBSEC and *L. lactic* subsp. *lactis* was also prevented by the phage inoculation (Fig. 10). A study reported that the phage which was efficient in reducing biofilms had a broader host range than those infecting bacterial cells themselves^37^, thereby suggesting that the newly-isolated SBSEC phages will have a strong potential to control biofilm formation of more diverse bacterial species in the rumen. Therefore, isolated SBSEC phages could be recognized as those with anti-biofilm potential and efficacy in preventing lactic acid-producing bacteria biofilms. Based on the genomes of the newly isolated phages, phage-derived depolymerase has not been directly identified as an enzyme that specifically controls bacterial biofilm formation. However, tail proteins encoded in the genomes of the phages vB_SbRt-pBovineB21 (ORF 14 and ORF 16) and vB_SbRt-pBovineS21 (ORF 13 and ORF 16) may be putative depolymerase, which has been found in the tail fiber protein or tail spike protein^38,39^. Accordingly, further studies are warranted to investigate the detailed mechanisms of the anti-biofilm activity of the phages in our future analysis. This is, to the best of our knowledge, the first study that reports the biological and genetic properties of lytic phages infecting SBSEC representatives, thus resulting in a potential to control ruminal microbiota (Table 2).

## Conclusions

This study provides biological and genetic information on two novel lytic phages (vB_SbRt-pBovineB21 and vB_SbRt-pBovineS21) infecting various species (or subspecies) of the ruminal SBSEC. Based on their morphological and genetic properties, both phages can be assigned as new species of *Fischettivirus*. In addition, comparative genome analyses revealed their uniqueness when compared with other known *Streptococcus* phages. The broad lytic activity of the isolated phages revealed their potential as biocontrol agents against SBSEC and lactic acid-producing bacteria that cause metabolic disorders in animals and humans. To summarize, the newly isolated phages can improve the safety and stability of ruminal microbiota as alternative biocontrol agents.

## Materials and methods

### Bacterial strains

In total, 59 SBSEC strains (51 isolates and 8 strains), *S. agalactiae* KCCM 11957^ T^, *Lactobacillus plantarum* subsp. *plantarum* KCTC 3108^ T^, *Lactobacillus sakei* KCCM 40264^ T^, *Lactobacillus casei* KCCM12452^T^, and *Lactococcus lactis* subsp. *lactis* KCCM 41572^ T^ were used in this study, as listed in Table 1. Among the 59 SBSEC strains used in this study, 51 were previously identified as SBSEC members based on *sodA* gene sequence analysis and their potential genetic diversity has been determined^21^. All the strains were cultured in tryptic soy agar (TSA; Difco, USA) at 37 °C for 24 h and stored in tryptic soy broth (TSB; Difco, USA) with 10% glycerol at -80 °C until use.

### Phage isolation and propagation

The host bacterial strain used for phage isolation in this study was *S. ruminicola* KCTC 43,306, which was recently reported as a novel SBSEC^3^. Samples were collected from bovine fecal and sewage to isolate the SBSEC phages. Briefly, the host strain grown in the exponential phase in TSB was co-cultivated with equal amounts of the collected samples at 37 °C for 24 h with shaking. The mixtures were centrifuged at 10,000 × *g* for 20 min and filtered with a 0.45 μm syringe filter (Millex, Merck Millipore Ltd., Ireland). Serial dilutions of the filtrate in distilled water were prepared and plated onto 0.7% TSB soft agar containing 1 mL culture of the host strain with an OD 600 nm of 0.3. This process was repeated at least three times to obtain single plaques. Thereafter, well-isolated phages were propagated using the conventional double-layered agar method^40^ and stored at 4 °C until use.

### Transmission electron microscopy

The morphology of the isolated SBSEC phages was examined using transmission electron microscopy. For negative staining, the propagated phage particles (approximately 10^9^ PFU/mL) were placed onto glow-discharged carbon-coated copper grids and allowed to absorb for 2 min at room temperature for negative staining. The samples were negatively stained with 2% (w/v) uranyl acetate (UrAc; Electron Microscopy Sciences, Inc., USA) solution for 1 min, followed by blotting off UrAc, and were then observed using a Bio-High voltage EM system (JEM-1400 Plus; JEOL Ltd., Japan) at 120 kV acceleration voltage in Korea Basic Science Institute (KBSI).

### Host range determination

To determine the host range of the isolated SBSEC phages, 60 *Streptococcus* spp., three strains of *Lactobacillus* sp., and one of *Lactococcus* sp. were tested using the double-layered agar method and spot assay. Briefly, 10 µL of phage lysates (approximately 10^9^ PFU/mL) was spotted onto a double-layer agar plate and added to 1 mL of the bacterial strain culture. After incubation at 37 °C for 24 h, the plate was observed for lysis zone formation.

### Adsorption and one-step growth curve

The adsorption assay was performed as previously described^41^. A 1:1 mixture of the host bacterial strain in the exponential phase (approximately 10^8^ CFU/mL) and phage lysates at an MOI of 0.0001 was incubated at 37 °C. Samples (100 µL) were collected at 0, 5, 10, 13, 15, 17, 20, and 25 min after phage inoculation and centrifuged at 12,000 × *g* at 4 °C for 5 min. To determine the proportion of free phages, the supernatants were cultured in triplicate using the double-layered agar method. A one-step growth assay was performed to determine the latent time and burst size, as previously described^41^. The phage lysates at an MOI of 0.0001 and the host strain at the log phase (approximately 10^8^ CFU/mL) were co-cultivated in equal amounts at 37 °C for 15 min, allowing adsorption to the host. The supernatant was removed after centrifugation at 12,000 × *g* and 4 °C for 5 min. The remaining pellets were re-suspended in 10 mL of pre-heated TSB and incubated at 37 °C with shaking. Suspensions (100 µL) were collected at 0, 5, 10, 15, 20, 25, 30, 40, 50, 60, 70, 80, 90, and 100 min and immediately diluted with distilled water for the double-layered agar method in triplicate.

### Thermal and pH stability

For the thermal stability test of the isolated SBSEC phages, 100 µL of phage lysate (approximately 10^8^ PFU/mL) was mixed with 900 µL of 0.1 M PBS (pH 7.0) and statically incubated at 4, 16, 25, 37, 45, 56, and 80 °C for 3 h. For the pH stability test, 100 µL of phage lysate (approximately 10^8^ PFU/mL) was added to a series of tubes containing 900 µL of pH buffer solution (Samchun Chemicals, Korea) at pH 2, 3, 4, 4.6, 5, 6, 7, 8, 9, 10, 11, and 12 and incubated at 4 °C for 3 h. After incubating all test samples, the phage titer was determined in triplicate using the double-layered agar method.

### Bacteriolytic activity assay

To evaluate the bacteriolytic effects of isolated SBSEC phages against the SBSEC, host cell lysis tests were performed as previously reported^42^. TSB (10 mL) was inoculated with the host strain and incubated at 37 °C until the log phase was reached. The culture was inoculated with phage lysates at MOIs of 0.001, 0.01, 0.1, 1, and 10 at 37 °C for 24 h with shaking, and the positive control was used as an MOI of 0. The absorbance was determined by measuring at OD 600 nm every 1 h for 10 h, which was performed in triplicate.

### Whole-genome sequencing and bioinformatic analysis

Total genomic DNA extracted by Macrogen (Seoul, Korea) was sequenced on the Illumina HiSeq X-10 platform (Illumina, USA) by constructing a DNA library using the TruSeq Nano DNA Library Prep Kit (Illumina, USA) and assembled using SPAdes (ver. 3.13.0) genome assembler^43^, using various k-mers. The putative ORFs of the genome were annotated using Rapid Annotation from the Subsystem Technology (RAST) server^44^. To determine sequence similarity, all predicted ORFs were analyzed using BLASTP^45^. Functionally conserved domains were compared with known proteins using the Pfam-A (ver. 35)^46^ and the RCSB PDB database^47^ through HHpred^48^. Transmembrane domains were predicted using TMHMM 2.0^49^, and signal peptides were analyzed using SignalP (ver. 6.0)^50^. PhageTerm^51^ was performed using the Galaxy server to determine phage termini and packaging mechanism. The genomes of the isolated SBSEC phages were screened for tRNAs using tRNAscan-SE 2.0^52^. Additionally, virulence and antibiotic resistance genes were detected using the Virulence Factors of Pathogenic Bacteria Database (VFDB)^53^ and Antibiotic Resistance Gene-ANNOTation (ARG-ANNOT)^54^.

Genome maps of isolated SBSEC phages were visualized using the CGView Server^55^. A phylogenetic tree based on the available genomes of isolated SBSEC phages with the *Rountreeviridae* family strains in GenBank was constructed using the Virus Classification and Tree Building Online Resource system^56^ and the Genome-BLAST Distance Phylogeny method^57^. The branch support of the tree was inferred using FastME (ver. 2.1.6.1)^58^ post-processing for the formulas D0. Amino acid sequences encoding major capsid protein and DNA polymerase were aligned using Clustal X (ver. 2.1)^59^ and BioEdit (ver. 7.1.0.3)^60^ and a single phylogenetic tree was constructed using the maximum likelihood (ML) method in MEGA X^61^. Bootstrap values were calculated for 100 replicates. A dot plot of genome sequences was generated using Gepard 2.1 using the default settings^62^. OrthoANI^63^ was used to calculate the average nucleotide identity (ANI) values of 30 phage strains belonging to the *Rountreeviridae* family in the GenBank database. A heat map based on the ANI values was generated using the R package heatmap (ver. 4.1.2)^64^. Comparative analysis of isolated SBSEC phages closely related to *Streptococcus* phage C1 was conducted using Easyfig^65^.

### Anti-biofilm activity

The efficacy of the isolated SBSEC phages in preventing biofilm formation by the host strain was assessed as previously reported^41^. TSB broth supplemented with 1% sucrose and 1% CaCl_2_ at a concentration of 0.1 M (TSBs-CaCl_2_) was used to perform these assays as it aids biofilm formation. Overnight cultured host strains (approximately 10^8^ CFU/mL) were diluted 1:100 with TSBs-CaCl_2_, and 100 µL aliquots of this diluted culture were mixed with phage lysate at different concentrations (10^5^, 10^6^, 10^7^, 10^8^, and 10^9^ PFU/mL) in equal amounts. The mixtures were added to each well of a sterile flat-bottomed 96-well plate (SPL Life Sciences, Korea) and incubated at 37 °C for 24 and 48 h. TSB without phage was the negative control. Next, the supernatant on the plates was washed with distilled water for three times to remove planktonic cells, and biofilms formed in each well were stained with 0.1% crystal violet (CV; Sigma, UK) for 20 min at room temperature. CV in each well was washed and dissolved in 100 µL of ethanol, and the OD was measured at 595 nm using a microplate reader (Spectramax 190, Molecular Devices, USA). CFUs were determined using serial dilutions of re-suspended distilled water to count viable bacterial cells in the biofilm. The efficacy of the isolated SBSEC phages in preventing biofilm formation by the other phage-susceptible strains were also evaluated using a single concentration of phage lysate (10^7^ PFU/mL) and the results were analyzed by incubation at 37 °C for 24 h.

### Confocal laser scanning microscope analysis

The biofilm prevention ability of the isolated SBSEC phages was observed by imaging using CLSM (LSM 800 META, Zeiss, Germany). Biofilm treated with each phage (10^6^ PFU/mL) when compared to the untreated phage was cultured in the sterile coverslips (Marienfeld-Superior, Germany) placed in non-surface treated six-well plates (SPL life sciences, Korea) at 37 °C for 24 and 48 h. The formed biofilms were stained with Syto 9 (BacLight, Thermo Fisher, USA) for 20 min in the dark. The stained biofilms in each well were mounted on the slide glass (Marienfeld-Superior, Germany). The live cell counts emitting green fluorescence were detected via excitation at 488 nm and emission at 498 nm and visualized using a 10 × objective using CLSM.

### Statistical analysis

Statistical analyses were conducted using GraphPad Prism 7 (GraphPad Software Inc., San Diego, CA, USA) to perform two-group comparisons using Student’s *t*-test and multi-group comparisons using two-way analysis of variance (ANOVA) with Tukey’s test. The significance level was set at p < 0.05 (*).

### Accession numbers of nucleotide sequences and strain deposition

The complete genome sequences of the isolated SBSEC phages have been deposited in GenBank under the accession numbers ON759209 (phage vB_SbRt-pBovineB21) and ON759210 (phage vB_SbRt-pBovineS21). SBSEC phage vB_SbRt-pBovineB21 was deposited in the KCCM under KCCM13031P, and vB_SbRt-pBovineS21 was deposited in the KCTC under KCTC 4834.

## Supplementary Information

Supplementary Information 1.

## Data Availability

All data generated or analyzed during this study are included in this published article and its supplementary information files. The complete genome sequences of the isolated SBSEC phages have been deposited in GenBank under the accession numbers ON759209 (phage vB_SbRt-pBovineB21) and ON759210 (phage vB_SbRt-pBovineS21).
