# Supplementary material for: Characterization of two lytic bacteriophages, infecting Streptococcus bovis/equinus complex (SBSEC) from Korean ruminant
Source: Sci Rep. 2023 Jun 5;13:9110. doi: 10.1038/s41598-023-36306-x (PMC10241823; doi:10.1038/s41598-023-36306-x)
Supplement: Supplementary file 1 — Supplementary Information 1. [file 41598_2023_36306_MOESM1_ESM.docx]

**Two lytic bacteriophages, vB_SbRt-pBovineB21 and vB_SbRt-pBovineS21, infecting *Streptococcus bovis/equinus* complex (SBSEC) from Korean ruminants: isolation, characterization, and genomic analysis**

Seon Young Park1,‡, Hyemin Kwon2,‡, Sang Guen Kim^3^, Se Chang Park^3^, Ji Hyung Kim4,^*^, Seongwon Seo1,^*^

1Division of Animal and Dairy Sciences, College of Agriculture and Life Science, Chungnam National University, Daejeon 34134, South Korea

2Department of Microbiology and Molecular Biology, College of Bioscience and Biotechnology, Chungnam National University, Daejeon 34134, South Korea

3Laboratory of Aquatic Biomedicine, College of Veterinary Medicine and Research Institute for Veterinary Science, Seoul National University, Seoul 08826, South Korea

4Department of Food Science and Biotechnology, College of Bionano technology, Gachon University, Seongnam 13120, South Korea

^‡^ These authors equally contributed to this work.

^*^Corresponding authors.

**Supplementary information**


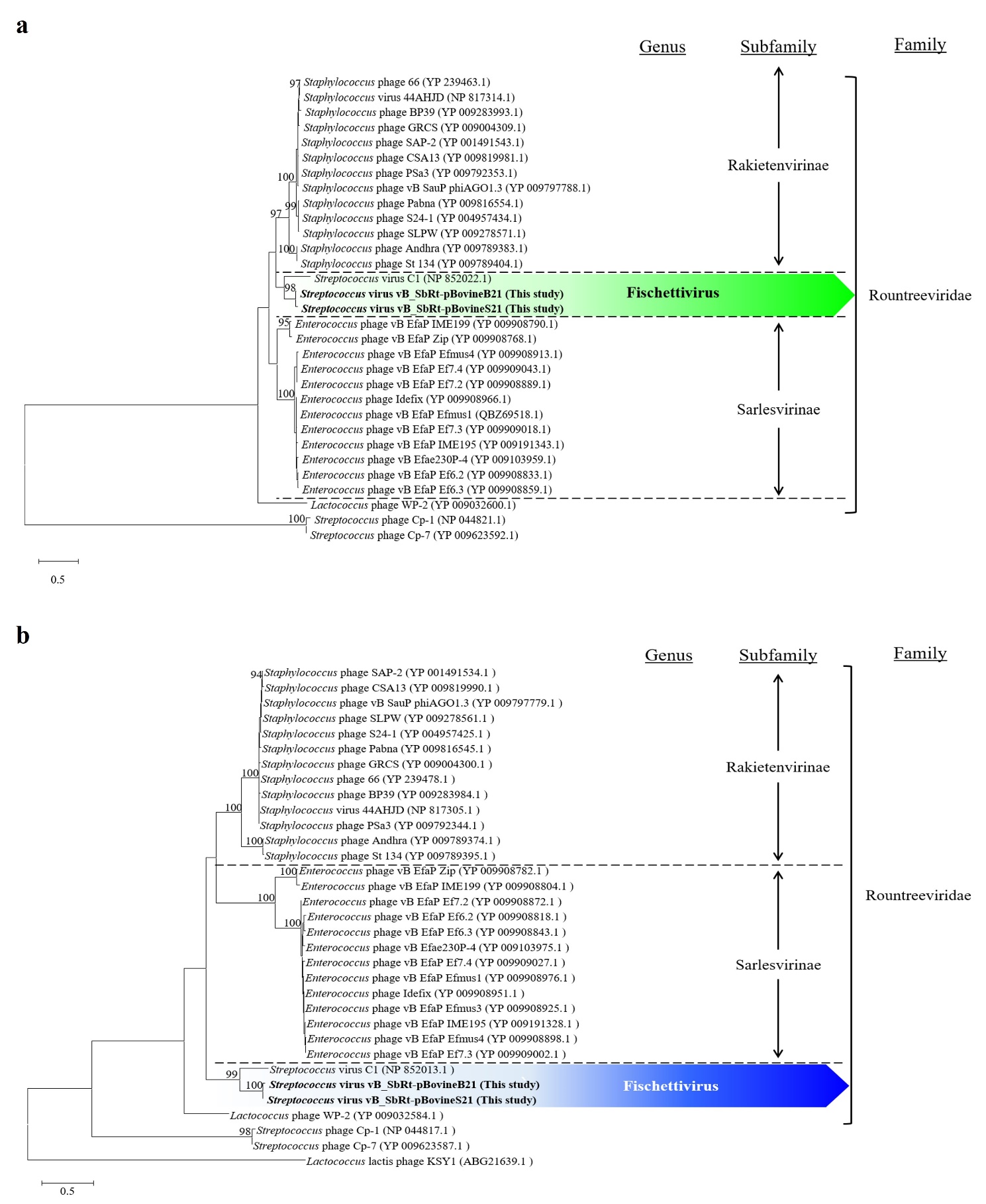


**Supplementary Fig. 1 Phylogenetic trees of the isolated SBSEC phages based on the major capsid protein and DNA polymerase**

The two trees were generated using the amino acid sequences encoding major capsid protein (a) and DNA polymerase (b) from the *Rountreeviridae* family. The green and blue arrows indicate the *Fischettivirus* genus.

**Supplementary Table 1. Functional grouping of predicted open reading frames (ORFs) and the homology of the SBSEC phage vB_SbRt-pBovineB21**

| Group | Gene product | | | | Putative function  [Conserved domain] | Best match virus  (E-value) | Identity  (%) | Predicted TMH  and signal peptide | |
| --- | --- | --- | --- | --- | --- | --- | --- | --- | --- |
|  | ORF  No. | Range | Strand | Length  (AA) |  |  |  | TMHHM | SignalP |
|  | 1 | 47-222 | + | 57 | No match | No match | - | 0 | N |
|  | 2 | 234-602 | + | 122 | Hypothetical protein | *Lactococcus* phage P1046 (2e-12) | 50.0 | 0 | N |
|  | 3 | 599-688 | + | 29 | No match | No match | - | 1 | N |
|  | 4 | 685-1212 | + | 175 | Hypothetical protein | *Streptococcus* phage C1 (0.06) | 25.5 | 0 | N |
|  | 5 | 1212-1364 | + | 50 | No match | No match | - | 0 | N |
|  | 6 | 1367-1645 | + | 92 | No match | No match | - | 0 | N |
| Nucleotide metabolism | 7 | 1707-2027 | + | 106 | Hypothetical protein [PF16773; Single-stranded DNA binding protein; *Lactococcus* phage p2] | *Streptococcus* phage C1 (1e-26) | 51.0 | 0 | N |
| Structure & packaging | 8 | 2066-3313 | + | 415 | Putative encapsidation protein [7JQP_A; Encapsidation protein; packaging motor; ATPase, motor protein; *Lactococcus* phage asccphi28] | *Streptococcus* phage C1 (2e-138) | 50.1 | 0 | N |
| Nucleotide metabolism | 9 | 3332-5650 | + | 772 | DNA polymerase [PF03175; DNA_pol_B_2; DNA polymerase type B, organellar and viral; *Streptococcus* phage Cp-1] | *Streptococcus* phage C1 (0.0) | 53.3 | 0 | N |
| Lysis | 10 | 5647-5988 | - | 113 | lil [PF13392; HNH_3; HNH endonuclease; Bacteriophage SPO1] | *Streptococcus* phage C1 (1e-21) | 50.5 | 0 | N |
|  | 11 | 5957-6169 | - | 70 | No match [4F87_A; PlyCB; lysin; antimicrobial protein; *Streptococcus* phage C1] | No match | - | 0 | N |
|  | 12 | 6162-6551 | - | 129 | Holin [PF05105; Phage_holin_4_; Bacteriophage holin family; *Streptococcus* phage Cp-1] | *Streptococcus* phage C1 (6e-11) | 38.7 | 2 | N |
|  | 13 | 6617-8044 | - | 475 | plyCA [PF18013; Phage_lysozyme2; Phage tail lysozyme; *Streptococcus* phage C1] | *Podoviridae* sp. (3e-11) | 40.2 | 0 | N |
| Tail structure | 14 | 8058-9809 | - | 583 | Phage tail protein [PF16838; Caud_tail_N; Caudoviral major tail protein N-terminus; *Streptococcus* phage C1] | *Streptococcus* phage C1 (6e-103) | 37.7 | 0 | N |
|  | 15 | 9887-10585 | - | 232 | Hypothetical protein | *Podoviridae* sp. (8e-35) | 35.0 | 0 | N |
| Tail structure | 16 | 10589-11533 | - | 314 | Tail fiber protein [6IAB_A; Tail fiber; structural protein; receptor binding protein; *Staphylococcus* phage P68] | *Podoviridae* sp. (4e-57) | 38.5 | 0 | N |
|  | 17 | 11547-12206 | - | 219 | Head to tail adaptor [6Q3G_BC; Lower collar protein; structural protein; receptor binding protein; *Staphylococcus* phage P68] | *Podoviridae* sp. (4e-30) | 38.2 | 0 | N |
| Structure & packaging | 18 | 12206-13168 | - | 320 | Putative upper collar protein [PF05352; Phage_connector; Phage Connector (GP10); *Staphylococcus* phage P68] | *Streptococcus* phage C1 (6e-77) | 45.5 | 0 | N |
|  | 19 | 13257-14411 | - | 384 | Major capsid protein [6IAT_A; Major head protein; structural protein; *Staphylococcus* phage P68] | *Streptococcus* phage C1 (1e-179) | 63.6 | 0 | N |
|  | 20 | 14411-14572 | - | 53 | Hypothetical protein [6IAT_E; Arstotzka protein; structural protein; *Staphylococcus* phage P68] | *Enterococcus* phage vB_EfaP_Efmus3 (7e-04) | 61.3 | 0 | N |
|  | 21 | 14585-14881 | - | 98 | No match | No match | - | 0 | N |
|  | 22 | 14898-15040 | - | 46 | No match | No match | - | 0 | N |
|  | 23 | 15342-15511 | - | 55 | No match | No match | - | 0 | N |
|  | 24 | 15511-15642 | - | 43 | No match | No match | - | 1 | N |
|  | 25 | 15762-15923 | - | 53 | No match | No match | - | 0 | N |
|  | 26 | 15920-16078 | - | 52 | No match | No match | - | 0 | N |
|  | 27 | 16073-16197 | - | 40 | No match | No match | - | 0 | N |

**Supplementary Table 2. Functional grouping of predicted open reading frames (ORFs) and the homology of the SBSEC phage vB_SbRt-pBovineS21**

| Group | Gene product | | | | Putative function  [Conserved domain] | Best match virus  (E-value) | Identity  (%) | Predicted TMH  and signal peptide | |
| --- | --- | --- | --- | --- | --- | --- | --- | --- | --- |
|  | ORF  No. | Range | Strand | Length  (AA) |  |  |  | TMHHM | SignalP |
|  | 1 | 181-405 | + | 74 | No match | No match | - | 0 | N |
|  | 2 | 418-785 | + | 121 | Hypothetical protein | *Lactococcus* phage CHPC971 (2e-12) | 50.0 | 0 | N |
|  | 3 | 866-1393 | + | 175 | Hypothetical protein | *Streptococcus* phage C1 (0.015) | 25.5 | 0 | N |
|  | 4 | 1393-1548 | + | 51 | No match | No match | - | 0 | N |
|  | 5 | 1545-1823 | + | 92 | No match | No match | - | 0 | N |
| Nucleotide metabolism | 6 | 1884-2204 | + | 106 | Hypothetical protein [PF16773; Single-stranded DNA binding protein; *Lactococcus* phage p2] | *Streptococcus* phage C1 (1e-28) | 51.9 | 0 | N |
| Structure & packaging | 7 | 2241-3490 | + | 415 | Putative encapsidation protein [7JQP_A; Encapsidation protein; packaging motor; ATPase, motor protein; *Lactococcus* phage asccphi28] | *Streptococcus* phage C1 (5e-126) | 52.1 | 0 | N |
| Nucleotide metabolism | 8 | 3509-5827 | + | 772 | DNA polymerase [PF03175; DNA_pol_B_2; DNA polymerase type B, organellar and viral; *Streptococcus* phage Cp-1] | *Streptococcus* phage C1 (0.0) | 54.0 | 0 | N |
| Lysis | 9 | 5824-6165 | - | 113 | lil [PF13392; HNH_3; HNH endonuclease; Bacteriophage SPO1] | *Streptococcus* phage C1 (1e-21) | 51.5 | 0 | N |
| Lysis  Tail structure | 10 | 6134-6346 | - | 70 | No match [4F87_A; PlyCB; lysin; antimicrobial protein; *Streptococcus* phage C1] | No match | - | 0 | N |
|  | 11 | 6339-6728 | - | 129 | Holin [PF05105; Phage_holin_4_; Bacteriophage holin family; *Streptococcus* phage Cp-1] | *Streptococcus* phage C1 (1e-10) | 38.7 | 2 | N |
|  | 12 | 6794-8221 | - | 475 | plyCA [PF18013; Phage_lysozyme2; Phage tail lysozyme; *Streptococcus* phage C1] | *Podoviridae* sp. (6e-12) | 39.1 | 0 | N |
|  | 13 | 8243-9994 | - | 583 | Phage tail protein [PF16838; Caud_tail_N; Caudoviral major tail protein N-terminus; *Streptococcus* phage C1] | *Streptococcus* phage C1 (2e-107) | 38.4 | 0 | N |
|  | 14 | 10064-10579 | - | 171 | Hypothetical protein | *Podoviridae* sp (6e-06) | 37.1 | 0 | N |
|  | 15 | 10625-11323 | - | 232 | Hypothetical protein | *Podoviridae* sp. (2e-36) | 35.5 | 0 | N |
| Tail structure | 16 | 11327-12271 | - | 314 | Tail fiber protein [6IAB_A; Tail fiber; structural protein; receptor binding protein; *Staphylococcus* phage P68] | *Podoviridae* sp. (4e-57) | 38.2 | 0 | N |
|  | 17 | 12294-12953 | - | 219 | Head to tail adaptor [6Q3G_BC; Lower collar protein; structural protein; receptor binding protein; *Staphylococcus* phage P68] | *Podoviridae* sp. (3e-29) | 37.3 | 0 | N |
| Structure & packaging | 18 | 12953-13915 | - | 320 | Putative upper collar protein [PF05352; Phage_connector; Phage Connector (GP10); *Staphylococcus* phage P68] | *Streptococcus* phage C1 (2e-77) | 45.8 | 0 | N |
|  | 19 | 13964-15118 | - | 384 | Major capsid protein [6IAT_A; Major head protein; structural protein; *Staphylococcus* phage P68] | *Streptococcus* phage C1 (7e-176) | 62.3 | 0 | N |
|  | 20 | 15118-15279 | - | 53 | Hypothetical protein [6IAT_E; Arstotzka protein; structural protein; *Staphylococcus* phage P68] | *Streptococcus* phage C1 (3.1) | 47.7 | 0 | N |
|  | 21 | 15292-15582 | - | 96 | No match | No match | - | 0 | N |
|  | 22 | 15595-15719 | - | 56 | No match | No match | - | 0 | N |
|  | 23 | 15955-16109 | - | 50 | No match | No match | - | 1 | N |
|  | 24 | 16112-16243 | - | 43 | No match | No match | - | 1 | N |
|  | 25 | 16363-16524 | - | 53 | No match | No match | - | 0 | N |
|  | 26 | 16618-16911 | - | 97 | No match | No match | - | 0 | N |

**Supplementary Table 3. Best hits for two SBSEC phages to NCBI database based on the complete genome sequence using BLASTn**

| Description | Taxonomy | | Query coverage  (%) | E-value | Identity (%) | Accession No. | |
| --- | --- | --- | --- | --- | --- | --- | --- |
|  | Family | Genus |  |  |  |  |  |
| **vB_SbRt-pBovineB21** | | | | | | |  |
| *Streptococcus* phage C1 | Rountreeviridae | Fischettivirus | 2 | 8e-40 | 75.6 | NC_004814.1 | |
| *Enterococcus* phage vB_EfaP_Ef6.3 | Rountreeviridae | Copernicusvirus | 1 | 5e-17 | 77.6 | NC_049933.1 | |
| **vB_SbRt-pBovineS21** | | | | | | |  |
| *Staphylococcus* phage BP39 | Rountreeviridae | Rosenblumvirus | 3 | 2e-36 | 72.5 | NC_031046.1 | |
| *Staphylococcus* phage SA1-CTA1 | Rountreeviridae | Rosenblumvirus | 1 | 1e-32 | 77.3 | MK922546.1 | |
| *Streptococcus* phage C1 | Rountreeviridae | Fischettivirus | 1 | 6e-31 | 77 | NC_004814.1 | |
| *Staphylococcus* phage vB_ScaM-V1SC05 | Rountreeviridae | Andhravirus | 1 | 2e-26 | 76.1 | OP297179.1 | |
| *Staphylococcus* phage SeAlphi | Rountreeviridae | Andhravirus | 1 | 2e-26 | 76.1 | NC_070880.1 | |
| *Staphylococcus* phage Andhra | Rountreeviridae | Andhravirus | 1 | 8e-20 | 74.6 | NC_047813.1 | |
| *Staphylococcus* phage vB_SurP-PSU3 | Rountreeviridae | Andhravirus | 1 | 4e-18 | 74.4 | OK574338.1 | |
| *Enterococcus* phage vB_EfaP_Ef6.3 | Rountreeviridae | Copernicusvirus | 0 | 1e-07 | 85.9 | NC_049933.1 | |

**Supplementary Table 4. Genomic information of the *Rountreeviridae* family based on the classification by ICTV**

| Classification | | | Name of phage | Host strains | Genome size  (bp) | Genome  type | Genbank  No. |
| --- | --- | --- | --- | --- | --- | --- | --- |
| Family | Subfamily | Genus |  |  |  |  |  |
| Rountreeviridae |  | Fischettivirus | vB_SbRt-pBovinveB21 | *Streptococcus ruminicola* KCTC 43306^T^ | 16,260 | Linear | This study |
|  |  |  | vB_SbRt-pBovinveS21 | *Streptococcus ruminicola* KCTC 43306^T^ | 17,280 | Linear | This study |
|  |  |  | C1 | *Streptococcus* sp. 'group C' | 16,687 | Linear | NC_004814 |
|  |  | Negarvirus | WP2 | *Lactococcus garvieae* | 18,899 | Linear | NC_024149 |
|  | Rakietenvirinae | Andhravirus | Andhra | *Staphylococcus epidermidis* RP62A | 18,546 | Linear | NC_047813 |
|  |  |  | St134 | *Staphylococcus epidermidis* CEMTC 2044 | 18,275 | Linear | NC_047814 |
|  |  | Rosenblumvirus | rv44AHJD | *Staphylococcus* sp. | 16,784 | Linear | NC_004678 |
|  |  |  | GRCS | *Staphylococcus aureus* NRS382 | 17,869 | Linear | NC_023550 |
|  |  |  | AGO13 | *Staphylococcus aureus* | 17,603 | Linear | NC_047919 |
|  |  |  | PSa3 | *Staphylococcus aureus* | 17,602 | Linear | NC_047855 |
|  |  |  | SAP2 | *Staphylococcus aureus* | 17,938 | Linear | NC_009875 |
|  |  |  | SLPW | *Staphylococcus aureus* | 17,861 | Linear | NC_031008 |
|  |  |  | rv66 | - | 18,199 | Linear | NC_007046 |
|  |  |  | BP39 | *Staphylococcus aureus* | 17,641 | Linear | NC_031046 |
|  |  |  | CSA13 | *Staphylococcus aureus* | 17,034 | Linear | NC_048159 |
|  |  |  | pabna | *Staphylococcus aureus* RN4220 | 17,700 | Linear | NC_048107 |
|  |  |  | portland | *Staphylococcus aureus* | 17,711 | Linear | NC_055814 |
|  |  |  | SA46CL1 | *Staphylococcus aureus* | 17,508 | Linear | NC_055802 |
|  |  |  | SCH1 | *Staphylococcus aureus* | 18,023 | Linear | NC_047788 |
|  |  |  | LSA2366 | *Staphylococcus aureus* ATCC 25923 | 17,056 | Linear | NC_055916 |
|  |  |  | EBHT | *Staphylococcus aureus* | 17,471 | Linear | NC_055906 |
|  |  |  | S241 | *Staphylococcus aureus* SA27 | 18,168 | Linear | NC_016565 |
|  | Sarlesvirinae | Copemicusvirus | Efmus1 | *Enterococcus faecalis* Sorialis | 17,927 | Linear | NC_049938 |
|  |  |  | Efmus3 | *Enterococcus faecalis S*orialis | 18,286 | Linear | NC_049936 |
|  |  |  | Efmus4 | *Enterococcus faecalis* Sorialis | 18,186 | Linear | NC_049935 |
|  |  |  | Efae230P4 | *Enterococcus faecium* | 17,972 | Linear | NC_025467 |
|  |  |  | Ef62 | *Enterococcus faecalis* AH6 | 17,966 | Linear | NC_049932 |
|  |  |  | Ef63 | *Enterococcus faecalis* AH6 | 18,136 | Linear | NC_049933 |
|  |  |  | Ef72 | *Enterococcus faecalis* AH7 | 18,737 | Linear | NC_049934 |
|  |  |  | Ef73 | *Enterococcus faecalis* AH7 | 18,818 | Linear | NC_049939 |
|  |  |  | Ef74 | *Enterococcus faecalis* AH7 | 18,415 | Linear | NC_049940 |
|  |  |  | AE417 | *Enterococcus faecalis* ZEF2 | 18,477 | Linear | NC_055866 |
|  |  |  | Idefix | - | 18,168 | Linear | NC_049937 |
|  |  |  | IME195 | *Enterococcus faecalis* 003 | 18,655 | Linear | NC_028693 |
|  |  | Minhovirus | zip | *Enterococcus faecium* C410 | 18,742 | Linear | NC_049930 |
|  |  |  | IME199 | *Enterococcus faecium* | 18,838 | Linear | NC_049931 |
| Salasmaviridae |  | Cepunavirus | Cp1 | *Streptococcus pneumoniae* | 19,343 | Linear | NC_001825 |
|  |  |  | Cp7 | *Streptococcus pneumoniae* | 19,741 | Linear | NC_042114 |
